# Age-specific association between non-HDL-C and arterial stiffness in the Chinese population

**DOI:** 10.3389/fcvm.2022.981028

**Published:** 2022-09-26

**Authors:** Jie Wang, Rujia Miao, Zhiheng Chen, Jiangang Wang, Hong Yuan, Jing Li, Zheng Huang

**Affiliations:** ^1^Clinical Research Center, The Third Xiangya Hospital, Central South University, Changsha, China; ^2^Department of Cardiology, The Third Xiangya Hospital, Central South University, Changsha, China; ^3^Health Management Center, The Third Xiangya Hospital, Central South University, Changsha, China; ^4^Department of Rehabilitation, The Second Xiangya Hospital of Central South University, Changsha, China; ^5^Department of Neurosurgery, Xiangya Hospital, Central South University, Changsha, China

**Keywords:** PWV, non-HDL-C, arterial stiffness, vascular health, age-specific

## Abstract

**Background:**

While some epidemiological studies have found correlations between non-high-density lipoprotein cholesterol (non-HDL-C) and arterial stiffness, there are still exist controversial and age-stratified analysis are scarce yet.

**Methods:**

All individuals in this study were recruited in the Third Xiangya Hospital of Central South University from 2012 to 2016. Arterial stiffness was defined as brachial-ankle pulse wave velocity (baPWV) ≥1,400 cm/s. Association between non-HDL-C and arterial stiffness were explored using Cox proportional-hazards model. We also conducted subanalysis stratified by age. Furthermore, restricted cubic splines were used to model exposure-response relationships in cohort sample.

**Results:**

This cohort study included 7,276 participants without arterial stiffness at baseline. Over a median follow-up of 1.78 years (IQR, 1.03–2.49), 1,669 participants have identified with incident arterial stiffness. In multivariable-adjusted analyses, higher non-HDL-C concentration was associated with incident arterial stiffness with an adjusted hazard ratio (HR) of 1.09 [95% confidence interval (CI), 1.02–1.17] per 1 mmol/L increase. Compared with the lowest tertile, the HR for arterial stiffness with respect to the highest tertile of non-HDL-C was 1.26 (95% CI, 1.07–1.48). The results were similar in the analysis of young participants (age <60 years).

**Conclusion:**

Our study identified that non-HDL-C as a potential risk factor of arterial stiffness, especially for younger. The clinical benefits of decreasing non-HDL-C concentration should be further considered in the future.

## Introduction

Dyslipidemia have received increasing attention as the global burden of cardiovascular disease increases. A high serum cholesterol level has been shown to be a risk factor for cardiovascular disease (1, 2), with studies showing that the average cholesterol level is rising in Asian countries, which is of particular concern in China because the major rise in the Chinese population is in non-HDL-C (3). Notably, non-HDL-C is considered to have greater potential for cardiovascular disease (CVD) prognosis (4–6), and a stronger association with major CVD events among statin-treated patients compared with LDL-C (7). AHA/AHC and ESC/EAS guidelines have both recommended non-HDL-C for CVD risk estimation in 2019 (8, 9). However, it is unclear through which mechanism the effect of non-HDL-C on CVD is mediated.

Arterial stiffness plays a key role in CVD and mortality (10–12), and is recognized as a core characteristic of vascular aging. Pulse wave velocity is a commonly used method to measure arterial stiffness due to its advantages of convenience and non-invasiveness. In high-risk individuals, a 1 m/s increase in branchial-ankle pulse wave velocity (baPWV) raises the risk of a cardiovascular event by 12% (13). Currently, although most studies have reported that non-HDL-C is significantly correlated with PWV in young and old populations (6, 14–18), the clinical significance of non-HDL-C for arterial stiffness still remains controversial in the current available studies. Vallée et al. found a strong association between non-HDL-C and arterial stiffness (15), whereas a Chinese study showed inconsistent results in middle-aged and elderly people (17). Therefore, more studies are needed to verify the relationship between non-HDL-C and arterial stiffness in Chinese people, so as to explore whether the effect of non-HDL-C on CVD is mediated by vascular aging mechanism. In addition, no studies have evaluated the effects of non-HDL-C on arterial stiffness among different age groups.

Our research was conducted on a large-scale Chinese population. The purpose of this study was to examine the associations between non-HDL-C and arterial stiffness, and to simultaneously explore whether the effects of non-HDL-C were differed in different age group.

## Methods

### Study population

All individuals in this study were recruited in the Third Xiangya Hospital of Central South University between 2012 and 2016. Individuals lacking baPWV and non-HDL-C data, or with ineligible baPWV data, or <18 years of age were excluding. We included 67,116 participants with totally 84,853 person-exams. Furthermore, participants that underwent only one baPWV measurement were excluded in the study, excluding individuals with arterial stiffness at baseline (Supplementary Figure S1). The remaining individuals were comprised a cohort and remained for analyzed. Detailed information about the subjects can be found in our previous study (19). This cohort study is reported following the Strengthening the Reporting of Observational Studies in Epidemiology (STROBE) Statement (Supplementary Table S1).

### Clinical and laboratory assessments

Each participant completed a standardized health examination and a detailed questionnaire. Age, sex, height, and weight were recorded directly, and exercise, smoking, and drinking status were derived from the questionnaire. Physical activity was recorded as “Yes” if the subject reported exercising. Smoking was recorded as “Yes” if the subject reported smoking more than one cigarette per day on average. Drinking was recorded as “Yes” if the subject reported alcohol (beer, wine, or liquor) consumption at least two days per week on average.

Height and weight were measured in a standing position after having taken off shoes and clothes. BMI (body mass index) was calculated as weight (kg) divided by height (m) squared (i.e., kg/m^2^). Systolic blood pressure (SBP) and diastolic blood pressure (DBP) were measured with an electronic sphygmomanometer (Omron 9020) on the right upper arm. SBP and DBP were recorded as the average of two readings in the sitting position after a 10-min rest. If the two readings differed by >5 mmHg, a third measurement was performed and the average of all three readings was recorded.

Venous blood samples were collected after an overnight fast and then transferred into EDTA-containing vacuum tubes. Concentration of total cholesterol (TC), high density lipoprotein cholesterol (HDL-C), low density lipoprotein cholesterol (LDL-C) triglycerides (TG), and fasting blood glucose (FBG) were immediately analyzed at the clinical laboratory of the Third Xiangya Hospital with enzymatic methods (Hitachi 7600-110; Hitachi, Tokyo, Japan). Non-HDL-C was calculated as TC minus HDL-C.

### Measurement of baPWV

An automatic waveform analyzer (BP-203 RPE, Omron Healthcare, Dalian, China) was used to measure baPWV and ankle-branchial index (ABI) simultaneously [details could be found in our previous study (19)]. In brief, after 5 min of rest, one cuff was wrapped around each arm and ankle, after which the analyzer obtained a report including the baPWV and ABI of the left and right sides of the body. The baPWV was measured twice on both sides of the body, with the average recorded as the final value. An ABI <0.9 was considered to indicate severe peripheral arterial disease, which might lead to measurement error (20). To decrease measurement bias, subjects with bilateral ABI <0.9 were excluded from analysis, and subjects with ABI <0.9 on one side of the body were only evaluated using the baPWV from the other side. Furthermore, subjects with an average difference in baPWV ≥1,000 cm/s on the left and right sides of the body were also excluded (21). The primary endpoint in this cohort study was incidence of arterial stiffness (defined as a individual with baPWV ≥1,400 cm/s). For individual with arterial stiffness, the endpoint time was defined as the time when arterial stiffness was first detected, and for people without arterial stiffness, endpoint time was defined as the time of the last valid measurement.

### Statistical analysis

Characteristics of participants were presented as the mean followed by the standard deviation (SD) in parentheses for continuous variables with normal distributions, or the median followed by the interquartile range (IQR) in parentheses for continuous variables with skewed distributions, or as percentages for categorical variables. Differences between groups were evaluated using the Kruskal–Wallis test for continuous variables, and the chi-square test for categorical variables. Two-tailed *P*-values of ≤0.05 were considered significant in all analyses. The Cox proportional-hazards model was applied to calculate the hazard ratio (HR) and 95% confidence interval (CI) of non-HDL-C for incident arterial stiffness with exposure both as a continuous variable (per 1 mmol/L increase) and as a categorical variable (tertiles). Model 1 was adjusted for age and sex. Model 2 was additionally adjusted for BMI, SBP, and fasting blood glucose. Model 3 was additionally adjusted for exercise, smoking status, and drinking status. We also performed analysis stratified by age (two categories: <60 years; ≥60 years). Additionally, restricted cubic splines were done to model the concentrations of non-HDL-C as a continuous variable for the different age groups (<60 and ≥60).

Furthermore, some sensitivity analyses were conducted to confirm the robust of our analysis: (1) excluding individuals with missing data; (2) excluding individuals with <1 year follow-up time.

All statistical analyses were performed using Stata software (version 16.0; StataCorp LLC, College Station, TX, USA) and R version 4.0.3 (The R Foundation for Statistical Computing).

## Results

### Study population

The cohort study consisted of 7,276 participants for analysis with median follow-up of 1.78 years, after excluding individuals with only one health exam. The median age of all subjects was 44 (IQR, 37–49) years, and 2,354 were female. Individuals with arterial stiffness tended to be older and to have higher BMI, SBP, DBP, FBG TG, TC, LDL-C, and non-HDL-C, and lower HDL-C levels than subjects without arterial stiffness. Additionally, individuals with arterial stiffness were more likely to be male, smoking, and drinking (Table 1).

**Table 1:** Baseline characteristic of cohort.

**Parameter**	**Total**	**Without arterial stiffness**	**With arterial stiffness**	***P*-Value**
	***n* = 7,276**	***n* = 5,607**	***n* = 1,669**	
Age (years)	44 (37–49)	42 (36–48)	48 (42–55)	< 0.001
Sex (female, %)	2,354 (32.35%)	2,021 (36.04%)	333 (19.95%)	< 0.001
baPWV (cm/s)	1,261.50 (1,181.50–1,329.50)	1,236.50 (1,161.00–1,307.00)	1,328.50 (1,273.50–1,367.50)	< 0.001
SBP (mmHg)	118 (110–128)	116 (108–124)	126 (118–134)	< 0.001
DBP (mmHg)	74 (68–82)	74 (68–80)	80 (74–88)	< 0.001
Body mass index (kg/m^2^)	24.19 (22.02–26.31)	23.94 (21.81–26.11)	25.09 (23.05–26.85)	< 0.001
FBG (mmol/L)	5.09 (4.75–5.46)	5.05 (4.72–5.41)	5.24 (4.87–5.69)	< 0.001
TG (mmol/L)	1.34 (0.92–2.05)	1.27 (0.89–1.95)	1.57 (1.09–2.36)	< 0.001
HDL cholesterol (mmol/L)	1.47 (1.23–1.76)	1.50 (1.25–1.78)	1.38 (1.17–1.66)	< 0.001
LDL cholesterol (mmol/L)	2.58 (2.09–3.12)	2.56 (2.07–3.10)	2.68 (2.18–3.18)	< 0.001
TC (mmol/L)	4.87 (4.32–5.49)	4.83 (4.28–5.44)	4.99 (4.44–5.60)	< 0.001
non-HDL cholesterol (mmol/L)	3.36 (2.76–3.99)	3.30 (2.70–3.93)	3.56 (3.00–4.14)	< 0.001
Smoking status
No	3,272 (62.62%)	2,582 (64.18%)	690 (57.40%)	< 0.001
Yes	1,953 (37.38%)	1,441 (35.82%)	512 (42.60%)	
Drinking status
No	3,056 (58.49%)	2,427 (60.33%)	629 (52.33%)	< 0.001
Yes	2,169 (41.51%)	1,596 (39.67%)	573 (47.67%)	
Exercise
No	1,476 (28.25%)	1,166 (28.98%)	310 (25.79%)	0.031
Yes	3,749 (71.75%)	2,857 (71.02%)	892 (74.21%)	

baPWV, brachial-ankle pulse wave velocity; SBP, systolic blood pressure; DBP, diastolic blood pressure; BMI, body mass index; FBG, fasting blood glucose; TG, Triglycerides; HDL, high density lipoprotein; LDL, low density lipoprotein; TC, total cholesterol; data were presented as median (interquartile range, IQR) for continuous variables and percentage for dichotomous variables.

### Longitudinal association between non-HDL-C and incident arterial stiffness

During the median follow-up of 1.78 year (range: 0.05 to 4.72 years; IQR: 1.03–2.49 years), 1,669 were diagnosed with arterial stiffness according to the definition baPWV ≥1,400 cm/s (Table 2). Compared with participants with the lowest tertile of non-HDL-C, the fully adjusted HRs for arterial stiffness risk of non-HDL were 1.26 (95%CI, 1.07–1.48) among those with the highest tertile of non-HDL-C with *P*_for − trend_ = 0.005 (in Model 3; Table 2). The fully adjusted HRs of arterial stiffness incidence risk for per 1 mmol/L increase in non-HDL-C were 1.09 (95% CI, 1.02–1.17, in Model 3) among total participants (Table 2). Stratified analysis by age and sex revealed that the risk of incident arterial stiffness was significantly higher among younger participants (<60 years; Table 2).

**Table 2:** The HRs of non-HDL-C concentration with the incidence risk of arterial stiffness.

**Stratified by**		**Categorical**			***P*-trend**	**Per 1.0 mmol/L↑**
		**Tertile 1**	**Tertile 2**	**Tertile 3**		
Total	Case/*N*	401/2,440	582/2,424	686/2,412	–	1,669/7,276
	Model 1	Reference	1.26 (1.11, 1.44)***	1.47 (1.29, 1.66)***	< 0.001	1.16 (1.10,1.22)***
	Model 2	Reference	1.18 (1.02, 1.36)*	1.30 (1.13, 1.49)***	< 0.001	1.10 (1.04,1.17)**
	Model 3	Reference	1.15 (0.98, 1.35)	1.26 (1.07, 1.48)**	0.005	1.09 (1.02,1.17)**
< 60 years	Case/*N*	312/2,307	509/2,307	601/2,290	–	1,422/6,904
	Model 1	Reference	1.30 (1.12,1.50)***	1.45 (1.26, 1.67)***	< 0.001	1.14 (1.08, 1.21)***
	Model 2	Reference	1.25 (1.07, 1.47)**	1.32 (1.13, 1.55)**	0.001	1.09 (1.03,1.17)**
	Model 3	Reference	1.23 (1.03, 1.49)*	1.30 (1.08,1.56)**	0.005	1.09 (1.01, 1.17)*
≥60 years	Case/*N*	89/133	73/117	85/122	–	247/372
	Model 1	reference	0.97 (0.71, 1.33)	1.36 (1.00, 1.85)*	0.047	1.19 (1.02, 1.38)*
	Model 2	reference	0.90 (0.64, 1.27)	1.09 (0.78, 1.53)	0.608	1.09 (0.93,1.28)
	Model 3	reference	0.83 (0.58, 1.21)	1.00 (0.69, 1.45)	0.998	1.02 (0.85, 1.22)

Model 1: adjusted by age and sex. Model 2: Model 1 + adjusted by BMI, SBP, and fasting glucose. Model 3: Model 2 + adjusted by smoking status, drinking status, and exercise. Non-HDL cholesterol category: tertile 1, < 2.98 mmol/L; tertile 2, 2.98–3.76 mmol/L; tertile 3, >3.76 mmol/L. Reference defined as tertile 1.

^*^P < 0.05.

^**^P < 0.01.

^***^P < 0.001.

**Figure 1:**
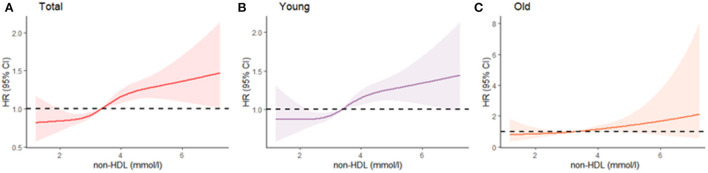
Age-specific continuous association of non-HDL-C and arterial stiffness **(A)** total; **(B)** young; **(C)** old. The Cox model used is adjusted for age and sex. Non-HDL-C was modeled using cubic splines. Median follow-up was 1.78 years.

The associations between non-HDL-C and risk of incident arterial stiffness across the entire levels were shown in Figure 1A. Additionally, stratified analyses revealed that the risk of incident arterial stiffness associated with non-HDL-C was differed among different age group (Figures 1B,C). The risk of incident arterial stiffness was significantly higher among younger participants (<60 years).

### Sensitivity analyses

The association of non-HDL-C with an increased risk of incident arterial stiffness was still robust in sensitivity analyses. After successively excluding the participants with missing data and participants with <1 follow-up year, the adjusted HRs of non-HDL-C for arterial stiffness were consistent with the results from the main analyses (Supplementary Table S3). The baseline characteristic of the remaining participants was presented in Supplementary Table S2. In the sensitivity analysis after excluding individuals with less than 1 year follow-up, the results still robust (Supplementary Table S3).

## Discussion

In this cohort study, our results showed that higher non-HDL-C concentration was associated with incidence risk of arterial stiffness. The effect of non-HDL-C was still robust in younger participants (<60 years old), while disappeared in older participants (≥60 years old). These findings may provide a clue that the potential target threshold of non-HDL-C and the intensity of lipid lowering should vary with age in future management decisions.

Arterial stiffness plays a central role in the vascular aging process of CVD. Furthermore, considering the strong correlation between the two, non-HDL-C may contribute to CVD through vascular aging. Previous studies, most of which were cross-sectional, reported that non-HDL-C significantly correlated with PWV in both the young and old (6, 14–18). However, these studies did not compare the effects of non-HDL-C on arterial stiffness between young and old subjects. In contrast to the previous studies, our study established a cohort to clarify the role of non-HDL-C, and to specifically compare its potentially different effects on younger and older subpopulations. We also used arterial stiffness defined by baPWV ≥1,400 cm/s as the dichotomous outcome. Our study further highlights the significant association between non-HDL-C and arterial stiffness. Surprisingly, non-HDL-C has different effects in different age groups. Non-HDL-C shows the strongest correlation with arterial stiffness in the young age group (<60). For the oldest age group (≥60), the effect disappeared altogether.

Non-HDL-C, including LDL, VLDL, lipoprotein(a), apolipoprotein B, and other effective components, has become a superior surrogate marker for treatment assessment. A meta-analysis including 233,455 subjects suggests that non-HDL-C is a more effective target for lipid-lowering therapy than LDL-C (22). Compared with LDL-C, non-HDL-C is also more strongly associated with atherosclerosis than LDL-C is (23). Furthermore, several studies have demonstrated that non-HDL-C is a better predictor of cardiovascular disease (4, 24). The Framingham Heart Study have found long term exposure to elevated non-HDL-C increases atherosclerotic cardiovascular disease (ASCVD) risk and mortality (5). Even for populations with low estimated 10-year ASCVD risk, non-HDL-C ≥160 mg/dl was associated with an 80% increased relative risk of CVD mortality (25). Patients with diabetes, metabolic syndrome, or obesity are also more likely to show elevated non-HDL-C in the presence of normal LDL-C, making non-HDL-C a better risk predictor for CVD (26, 27). In addition, non-HDL-C is calculated as TC minus HDL-C, without any additional cost or the need for prior fasting (28). Due to its convenience and greater predictive power, there are many guidelines, as well as a growing medical consensus, recommending non-HDL-C for clinical use (8, 9, 27). In mechanisms, dyslipidemia, especially a high level of non-HDL-C, was closely related to endothelial dysfunction (29). The important components of Non-HDL-C such as LDL and ApoB can cross the endothelial barrier and infiltrates specific areas of the arterial wall (30). Oxidative stress and chronic inflammation induced by long term exposure to high level of non-HDL also considered potential pathophysiological mechanisms in arterial stiffness. On the one hand, vascular inflammation causes arterial stiffness by stimulating proliferation of fibroblasts and smooth muscle cell (31, 32); on the other hand, inflammation and oxidative stress exacerbate endothelial dysfunction and impair arterial mechanical properties (33). These will ultimately lead to vascular aging and an increase in PWV.

It is necessary to discuss the age dependent association between non-HDL-C and arterial stiffness. As our study demonstrates, arterial stiffness is more often attributed to non-HDL-C in the young than in the elderly. Thus, young people are more likely to benefit from controlling non-HDL-C. For the elderly, non-HDL-C is not associated with arterial stiffness, further illustrating the etiological complexity of aging. Across life course, middle age was in the essential stage of arterial stiffness with a steeper increase in baPWV during this stage (19). This partly explains why non-HDL is more effective for participants <60. This age-specific effect may provide better guidelines for disease prevention and control. Our results depict a different risk curve for non-HDL-C concentration among the young and old, respectively (Figure 1). Therefore, the application of appropriate thresholds for different age groups should yield better outcomes. Our findings also advocate that the prevention of arterial stiffness should be initiated as early as possible to reduce the lifetime risk of cardiovascular disease

There are several advantages in our study. First, we have the strength of a large number of participants. Second, we discovered effects of non-HDL-C among different age group. Participants were divided into two groups according to age. The study provides the novel findings that non-HDL-c performs more effectively in identifying individuals at increased arterial stiffness risk especially for young individuals. However, several limitations remain. First, arterial stiffness is a chronic process, while the follow-up time of participants in our study was short. To reduce the influence of the short follow-up time, we did a sensitivity analysis among participants with more than 1 year follow-up. Our results still indicated the adverse effect of non-HDL-C. Second, our analysis in final model only included a subset of the participants due to missing values. To addressed this issue, we limited the main analysis to participants with complete data in sensitivity analysis. Third, our study lacks the information about lipid lowering therapy, that may be related with the risk of arterial stiffness. Finally, the included participants were Chinese and most of these participants were come from central region of China, which may be limited in the generalizability of these results.

In conclusion, our study indicates that non-HDL-C carries a greater incidence risk for arterial stiffness, especially for younger individuals. Our study suggests that the target threshold for non-HDL-C should be different according to the age.

## Data availability statement

The data analyzed in this study is subject to the following licenses/restrictions: Privacy of participants. Requests to access these datasets should be directed at: ZH, hzter1985@163.com.

## Ethics statement

The studies involving human participants were reviewed and approved by Institutional Review Board of The Third Xiangya Hospital of Central South University. The Ethics Committee waived the requirement of written informed consent for participation.

## Author contributions

Conceptualization: ZH and JL. Data curation: ZC. Formal analysis: JieW and RM. Investigation: JiaW and HY. Supervision: JL, RM, and ZH. Writing—original draft: JieW. Writing—review and editing: JL. All authors contributed to the article and approved the submitted version.

## Funding

The research was funded by the National Natural Science Foundation of China (82001274) and Natural Science Foundation of Hunan Province, China (2021JJ40941).

## Conflict of interest

The authors declare that the research was conducted in the absence of any commercial or financial relationships that could be construed as a potential conflict of interest.

## Publisher's note

All claims expressed in this article are solely those of the authors and do not necessarily represent those of their affiliated organizations, or those of the publisher, the editors and the reviewers. Any product that may be evaluated in this article, or claim that may be made by its manufacturer, is not guaranteed or endorsed by the publisher.
