# Supplementary material for: Age-specific association between non-HDL-C and arterial stiffness in the Chinese population
Source: Front Cardiovasc Med. 2022 Sep 26;9:981028. doi: 10.3389/fcvm.2022.981028 (PMC9548648; doi:10.3389/fcvm.2022.981028)
Supplement: Supplementary file 1 [file Data_Sheet_1.doc]

Table S1. Research checklist.

|  | Item No | Recommendation | Page  No |
| --- | --- | --- | --- |
| **Title and abstract** | 1 | (*a*) Indicate the study’s design with a commonly used term in the title or the abstract | 2 |
|  |  | (*b*) Provide in the abstract an informative and balanced summary of what was done and what was found | 2 |
| Introduction | | |  |
| Background/rationale | 2 | Explain the scientific background and rationale for the investigation being reported | 3 |
| Objectives | 3 | State specific objectives, including any prespecified hypotheses | 3 |
| Methods | | |  |
| Study design | 4 | Present key elements of study design early in the paper | 4 |
| Setting | 5 | Describe the setting, locations, and relevant dates, including periods of recruitment, exposure, follow-up, and data collection | 4 |
| Participants | 6 | (*a*) Give the eligibility criteria, and the sources and methods of selection of participants. Describe methods of follow-up | 3 |
|  |  | (*b*) For matched studies, give matching criteria and number of exposed and unexposed | NA |
| Variables | 7 | Clearly define all outcomes, exposures, predictors, potential confounders, and effect modifiers. Give diagnostic criteria, if applicable | 5 |
| Data sources/ measurement | 8* | For each variable of interest, give sources of data and details of methods of assessment (measurement). Describe comparability of assessment methods if there is more than one group | *4-5* |
| Bias | 9 | Describe any efforts to address potential sources of bias | 5 |
| Study size | 10 | Explain how the study size was arrived at | 6 |
| Quantitative variables | 11 | Explain how quantitative variables were handled in the analyses. If applicable, describe which groupings were chosen and why | 5 |
| Statistical methods | 12 | (*a*) Describe all statistical methods, including those used to control for confounding | 5 |
|  |  | (*b*) Describe any methods used to examine subgroups and interactions | 5 |
|  |  | (*c*) Explain how missing data were addressed | 5 |
|  |  | (*d*) If applicable, explain how loss to follow-up was addressed | 5 |
|  |  | (*e*) Describe any sensitivity analyses | 5 |
| Results | | |  |
| Participants | 13* | (a) Report numbers of individuals at each stage of study—eg numbers potentially eligible, examined for eligibility, confirmed eligible, included in the study, completing follow-up, and analysed | 6 |
|  |  | (b) Give reasons for non-participation at each stage | NA |
|  |  | (c) Consider use of a flow diagram | 4 |
| Descriptive data | 14* | (a) Give characteristics of study participants (eg demographic, clinical, social) and information on exposures and potential confounders | 6 |
|  |  | (b) Indicate number of participants with missing data for each variable of interest | 3 |
|  |  | (c) Summarise follow-up time (eg, average and total amount) | 6 |
| Outcome data | 15* | Report numbers of outcome events or summary measures over time | 6 |
| Main results | 16 | (*a*) Give unadjusted estimates and, if applicable, confounder-adjusted estimates and their precision (eg, 95% confidence interval). Make clear which confounders were adjusted for and why they were included | 6 |
|  |  | (*b*) Report category boundaries when continuous variables were categorized | Table2 |
|  |  | (*c*) If relevant, consider translating estimates of relative risk into absolute risk for a meaningful time period | NA |
| Other analyses | 17 | Report other analyses done—eg analyses of subgroups and interactions, and sensitivity analyses | 6 |
| Discussion | | |  |
| Key results | 18 | Summarise key results with reference to study objectives | 7 |
| Limitations | 19 | Discuss limitations of the study, taking into account sources of potential bias or imprecision. Discuss both direction and magnitude of any potential bias | 8 |
| Interpretation | 20 | Give a cautious overall interpretation of results considering objectives, limitations, multiplicity of analyses, results from similar studies, and other relevant evidence | 8 |
| Generalisability | 21 | Discuss the generalisability (external validity) of the study results | 8 |
| Other information | | |  |
| Funding | 22 | Give the source of funding and the role of the funders for the present study and, if applicable, for the original study on which the present article is based | 9 |

*Give information separately for exposed and unexposed groups.

Table S2 Baseline characteristic of included individuals after excluding missing data

| Parameter | Total | Without arterial stiffness | With arterial stiffness | P |
| --- | --- | --- | --- | --- |
|  | N=4599 | N=3587 | N=1012 |  |
| **age (years)** | 44 (37-49) | 43 (36-48) | 48 (43-56) | <0.001 |
| **sex (female,%)** | 1490 (32.40%) | 1290 (35.96%) | 200 (19.76%) | <0.001 |
| **baPWV (cm/s)** | 1256.00 (1175.00-1327.50) | 1230.5 (1152.00-1304.00) | 1328.25 (1276.50-1368.00) | <0.001 |
| **SBP (mmHg)** | 118 (110-128) | 116 (108-124) | 126 (118-134) | <0.001 |
| **DBP (mmHg)** | 74 (68-82) | 74 (68-80) | 80 (74-88) | <0.001 |
| **Body mass index (kg/m2)** | 24.16 (22.02-26.3) | 23.92 (21.80-26.08) | 25.11 (23.06-26.89) | <0.001 |
| **FBG (mmol/l)** | 5.08 (4.75-5.44) | 5.04 (4.72-5.39) | 5.23 (4.87-5.66) | <0.001 |
| **TG (mmol/l)** | 1.32 (0.91-2.03) | 1.27 (0.88-1.95) | 1.55 (1.08-2.36) | <0.001 |
| **HDL cholesterol (mmol/l)** | 1.47 (1.24-1.77) | 1.50 (1.25-1.79) | 1.40 (1.18-1.69) | <0.001 |
| **LDL cholesterol (mmol/l)** | 2.58 (2.09-3.12) | 2.56 (2.07-3.12) | 2.64 (2.17-3.14) | 0.064 |
| **TC (mmol/l)** | 4.87 (4.32-5.49) | 4.84 (4.28-5.47) | 4.94 (4.42-5.56) | <0.001 |
| **non-HDL cholesterol (mmol/l)** | 3.35 (2.74-3.99) | 3.31 (2.70-3.95) | 3.51 (2.95-4.13) | <0.001 |
| **Smoking status** |  |  |  |  |
| No | 2888 (62.80%) | 2888 (62.80%) | 576 (56.92%) | <0.001 |
| Yes | 1711 (37.20%) | 1711 (37.20%) | 436 (43.08%) |  |
| **Drinking status** |  |  |  |  |
| No | 2700 (58.71%) | 2172 (60.55%) | 528 (52.17%) | <0.001 |
| Yes | 1899 (41.29%) | 1415 (39.45%) | 484 (47.83%) |  |
| **Exercise** |  |  |  |  |
| No | 1272 (27.66%) | 1021 (28.46%) | 251 (24.80%) | 0.021 |
| Yes | 3327 (72.34%) | 2566 (71.54%) | 761 (75.20%) |  |

baPWV, brachial-ankle pulse wave velocity; SBP, systolic blood pressure; DBP, diastolic blood pressure; BMI, body mass index; FBG, fasting blood glucose; Triglycerides, TG; HDL, high density lipoprotein; LDL, low density lipoprotein; TC, total cholesterol; data were presented as median (interquartile range, IQR) for continuous variables and percentage for dichotomous variables.

Table S3 The HRs of non-HDL-C level with the incidence risk of arterial stiffness.

|  | **Categorical** | | | **P-trend** | **Per 1.0 mmol/l** ↑ |
| --- | --- | --- | --- | --- | --- |
|  | **Tertile 1** | **Tertile 2** | **Tertile 3** |
| **Excluding individuals with missing data^a^** | | | | |  |
| **Case/N** | 257/1543 | 358/1539 | 397/1526 | - | 1012/4599 |
| **Model 1** | reference | 1.25 (1.06, 1.47)** | 1.42 (1.21, 1.66)*** | <0.001 | 1.15 (1.08, 1.23)*** |
| **Model 2** | reference | 1.18 (1.01, 1.39)* | 1.30 (1.10, 1.53)** | 0.002 | 1.10 (1.03, 1.18)** |
| **Model 3** | reference | 1.18 (1.00, 1.39) | 1.28 (1.09, 1.51)** | 0.003 | 1.09 (1.02, 1.17)** |
|  |  |  |  |  |  |
| **Excluding individuals with <1 follow-up year^b^** | | | | |  |
| **Case/N** | 291/1965 | 439/1958 | 517/1961 | - | 1247/5884 |
| **Model 1** | reference | 1.36 (1.17, 1.58)*** | 1.52 (1.32, 1.76)*** | <0.001 | 1.16 (1.09, 1.23)*** |
| **Model 2** | reference | 1.26 (1.06, 1.50)** | 1.34 (1.13, 1.59)** | 0.001 | 1.10 (1.03, 1.18)** |
| **Model 3** | reference | 1.27 (1.05, 1.55)* | 1.35 (1.12, 1.64)** | 0.002 | 1.11 (1.03, 1.21)* |

Model 1: adjusted by age and sex; Model 2: Model 1 + adjusted by BMI, SBP, and fasting glucose; Model 3: Model 2 + adjusted by smoking status, drinking status, and exercise. Reference defined as tertile 1.

^a^ Non-HDL cholesterol category: tertile 1, < 2.96 mmol/l; tertile 2, 2.96 to 3.76 mmol/l; tertile 3, > 3.76 mmol/l. ^b^ Non-HDL cholesterol category: tertile 1, < 2.98 mmol/l; tertile 2, 2.98 to 3.74 mmol/l; tertile 3, > 3.74 mmol/l. * P<0.05, ** P<0.01, *** P<0.001.

**Online Figure 1**: Enrolment flow chart.

85,978 person-exams between 2012-2016

242 person-exams without baPWV or non-HDL-C measurements

29 person-exams with age <18 years old

854 person-exams did not pass baPWV quality check

84,853 person-exams enrolled

(67116 participants)

7,276 participants enrolled in cohort study

12220 participants with baPWV ≥ 1400 cm/s at baseline

54,896 participants with 1 visits
